# Coy Males and Seductive Females in the Sexually Cannibalistic Colonial Spider, *Cyrtophora citricola*

**DOI:** 10.1371/journal.pone.0155433

**Published:** 2016-06-01

**Authors:** Eric C. Yip, Na’ama Berner-Aharon, Deborah R. Smith, Yael Lubin

**Affiliations:** 1 Mitrani Department of Desert Ecology, Swiss Institute for Dryland Environmental and Energy Research, Jacob Blaustein Institutes of Desert Research, Ben-Gurion University of the Negev, Midreshet Ben-Gurion, Israel; 2 Department of Ecology and Evolutionary Biology, University of Kansas, Lawrence, KS, United States of America; Scientific Research Centre, Slovenian Academy of Sciences and Arts, SLOVENIA

## Abstract

The abundance of sperm relative to eggs selects for males that maximize their number of mates and for females that choose high quality males. However, in many species, males exercise mate choice, even when they invest little in their offspring. Sexual cannibalism may promote male choosiness by limiting the number of females a male can inseminate and by biasing the sex ratio toward females because, while females can reenter the mating pool, cannibalized males cannot. These effects may be insufficient for male choosiness to evolve, however, if males face low sequential encounter rates with females. We hypothesized that sexual cannibalism should facilitate the evolution of male choosiness in group living species because a male is likely to encounter multiple receptive females simultaneously. We tested this hypothesis in a colonial orb-weaving spider, *Cyrtophora citricola*, with a high rate of sexual cannibalism. We tested whether mated females would mate with multiple males, and thereby shift the operational sex ratio toward females. We also investigated whether either sex chooses mates based on nutritional state and age, and whether males choose females based on reproductive state. We found that females are readily polyandrous and exhibit no mate choice related to male feeding or age. Males courted more often when the male was older and the female was younger, and males copulated more often with well-fed females. The data show that males are choosier than females for the traits we measured, supporting our hypothesis that group living and sexual cannibalism may together promote the evolution of male mate choice.

## Introduction

The abundance of sperm relative to eggs generates inherent asymmetries between the sexes [1, 2]. Male reproduction is limited, not by their own sperm production, but by the eggs of their partners, and males can therefore increase their reproductive success by increasing their number of mates [3, 4]. While it is now widely accepted that polyandry may improve female fitness [5], the general rule remains that female fecundity is limited by their egg production and not by access to sperm [6]. Selection should favor males that invest in greater access to additional females, while females should select the highest quality males, thus leading to the patterns of male-male competition and female choosiness that have dominated sexual selection theory since Darwin [7].

Despite these general patterns, there are numerous examples of sex role reversal [8, 9, 10]. It was first proposed that mate choice evolved in conjunction with parental investment, so that the more choosy sex also invested more in each offspring [4]. While high paternal investment is sufficient for the evolution of male choosiness, it is not necessary, and there is growing evidence that males that invest little in their offspring are nevertheless choosy [11]. One alternative pathway to male choosiness is through the sexual cannibalism of males [12]. Females may reenter the mating pool, but cannibalized males are permanently removed, creating a more female-biased sex ratio. Males limited in their number of copulations by cannibalism cannot then fertilize all available females and may evolve mate choice [11]. However, a low sequential encounter rate with females can render high rates of sexual cannibalism inconsequential to male mate choice [13]. A male should not exercise choice if he is unlikely to encounter multiple females in his lifetime. Further, high rates of sexual cannibalism are predicted in species with high search costs and male-biased sex ratios [14, 15], traits that may select against male choosiness [13, 16]. We suggest that sexual cannibalism may be more likely to lead to male choosiness in group living organisms. If receptive females are clustered in space and time, then males may commonly encounter multiple receptive females simultaneously and face low search costs for alternative mates. When sexual cannibalism is frequent, males cannot increase their fitness by mating with multiple females, and selection should favor males that choose the highest quality females in the group.

**Fig 1:**
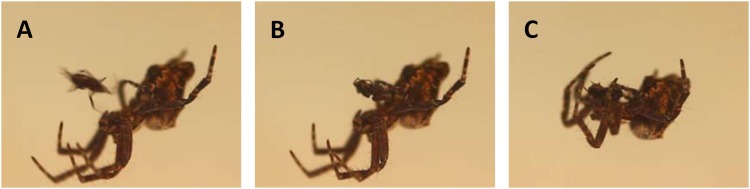
Copulation and sexual cannibalism in *C*. *citricola*. (A) shows the final approach of the male before he inserts his pedipalp (the male intromittent organ) into the genital opening of the female (B). The female bites the abdomen of the male while the pedipalp is still attached (C). The female pulls the male towards her mouthparts, and the pedipalp detaches. Copulation usually only lasts a few seconds.

We tested this hypothesis in the colonial orb weaving spider, *Cyrtophora citricola* Forsskål (Araneidae). This species preferentially builds webs in the presence of conspecific silk [17], forming dense colonies that can include thousands of individuals. Within the group, each spider spins a long-lasting horizontal orb web that lacks the sticky glue typical of other araneids. Like other colonial spider species, each *C*. *citricola* spider maintains and defends its own web within the shared framework of the colony, and, while spiders may benefit from group living, they do not cooperate in prey capture or brood care [18]. A previous study of *C*. *citricola* found that females cannibalized 100% of males that successfully copulated [19] (Fig 1). Males that either mature in a colony or immigrate into a colony will likely encounter multiple females and may therefore more readily evolve mate choice.

To test this prediction, we conducted three experiments. In the first, we tested the propensity for *C*. *citricola* females to mate with multiple males. If both males and females are monogamous, sexual selection should be relatively weak [20], and if females drop out of the mating population as fast as males, males may be unlikely to encounter multiple receptive females, regardless of social structure. In two additional experiments, we tested whether males choose females on the basis of reproductive state and nutritional state. First sperm precedence can severely limit paternity of subsequent males and thereby reduce the quality of mated females [21]. We examined male choice on the basis of reproductive state by measuring male preference for the webs of subadult, virgin adult, or mated adult females. Males might also choose more fecund females, and fecundity correlates with nutritional state [22]. We examined mate choice on the basis of nutritional state by recording courtship effort and copulation of *C*. *citricola* spiders placed on high and low feeding regimes. We also measured correlations between mating and spider age because age can reflect future mating opportunities [23] and fecundity in females [24].

## Materials and Methods

### Spider Collection and Rearing

We collected *C*. *citricola* from 6 locations in Israel for our three experiments. Three collection sites were along roads, which do not require collection permits for non-protected invertebrate species. In addition, we received permission to collect spiders from the director of the Beer Sheva Zoo, Dr. Haim Sivan, from the owner of Shuva Orchard, and from the Israel National Parks Authority (permit #40719) for the Bessor Nature Reserve. We collected immature spiders from Shuva Orchard (31°46′17″N 34°53′20″E) to test female re-mating and from Revivim (31°05′45″N 34°71′67″E) to examine male choice based on female reproductive state. Subadult females for these two experiments were housed in terrariums, while males and juveniles were kept in smaller cups (135 ml). Spiders were fed twice a week with *Drosophila melanogaster* or grasshopper nymphs (*Locusta migratoria*), according to spider size [25]. At maturity, the genital opening (epigynum) of females sclerotizes into a structure characteristic of the species, and we examined the epigynum of females after every molt to assess their maturity. Adult males were recognized by the complex structure of the pedipalp (the sperm delivery organ in spiders). There is extreme sexual size dimorphism in this species. Adult females weighed an average of 48.24 mg (± 15.37 S.D.) and averaged 7.17 mm (± 0.78 S.D.) in body length. Adult males weighed an average of 1.77 mg (± 0.56 S.D.) and averaged 2.33 mm (± 0.28 S.D.) in body length (see Fig 1 for relative sizes of the sexes).

For our experiment testing male choice based on female nutritional state, we collected egg sacs or gravid females from Revivim (31°05′45″N 34°71′67″E), the Arava Valley (30°43’07”N 35°16’54”E), the Bessor Reserve (31°13’48”N 34°30’29”E), the north shore of the Sea of Galilee (32°43’14”N 35°33’02”E), and the Beer Sheva Zoo (31°15’34”N 34°44’38”E). We separated juveniles into individual containers within 5 days of hatching and raised them to adulthood following the methods of Yip and Lubin [25]. After juveniles had molted twice, we randomly assigned them to high or low feeding regimes. We fed spiders on the low regime once a week and spiders on the high regime twice a week. Increased feeding translates to higher fecundity in female spiders, generally [22], and in *C*. *citricola*, specifically [25], and should provide the variance in female quality (reproductive potential) necessary to detect male choosiness. The feeding regime also influenced a variety of life history characteristics, including body size, development time, and longevity [25]. Adult females were placed into 1.4 L cylindrical containers. Each container contained 4 wooden poles to provide support for web construction. Spiders for all experiments were housed in the same climate-controlled room (26 C; 45% humidity; 12 hr light-dark cycle).

### Female re-mating

To test whether females would mate with more than one male, three groups of adult females (n = 20 for each group) were mated in the laboratory to record copulation success between August and September 2013. We paired the females with males again three days after the first mating in one group, ten days after mating in the second group, and the third group was not re-mated.

Males and females were paired randomly (all individuals were from the Shuva Orchard site). To begin the mating trial, we coaxed the male out of his web using soft forceps and allowed him to drop off the forceps on a dragline. We lowered the male onto the edge of the female’s orb web, which the male invariably grabbed, allowing us to remove the forceps and close the terrarium. We conducted trials in the same climate-controlled room in which spiders were reared and observed spiders for 2 hrs to record copulation.

### Male choosiness for female reproductive state

We tested for male preference of female reproductive state by measuring male affinity for the silk of mated, virgin, and subadult females. Males can detect the reproductive status of females by silk cues alone in several other spider species [26]. In the laboratory, we placed 23 large subadult, 27 virgin adult and 23 mated adult females in individual terrariums containing *Acacia* tree branches. The females were given ten days to construct their webs. After ten days, the terrariums were taken to an outdoor net house (10mX40m) with two rows of 1–1.5m high potted *Acacia* trees spaced three meters apart. We carefully removed each cluster of branches from the terrariums and placed each on the south-facing side of a tree, selected haphazardly. We removed the female from the web and replaced her with a male. We examined the trees for four days to check whether the male remained on the female’s web. The experiment took place between September and December 2012.

### Choosiness for mate’s nutritional state and age

Using spiders raised from eggs, we staged mating trials with virgin males and virgin females born from different clutches from a single population. Pairings were random, generating four feeding combinations: high females/high males, high females/low males, low females/high males, and low females/low males, where high and low indicate feeding regime. After introducing the male to the female terrarium, we recorded the behavior of both males and females for the first 30 mins of the trial, particularly courtship by both sexes and whether the pair copulated. Males perform discreet bouts of courtship, in which they lay silk parallel to the radial lines of the orb web and rhythmically strum the silk with their third legs. If the male stops courtship, he usually relocates to another part of the web and lays silk before courting again. We were thus able to count the number of male courtships in 30 mins. Female courtship is less distinct, but she also plucks the web with her third legs. She stops and starts plucking sporadically, making delineations of the distinct bouts of courtship impossible. We recorded whether the female engaged in courtship and the sex that initiated courtship first. We also recorded cannibalism, and, if the male survived the mating trial, we recorded the number of days before he disappeared from the female’s web. Because the probability of escape was extremely low, missing males were presumed eaten. We take the consumption of the male as evidence of copulation, as the rate of pre-copulatory sexual cannibalism is low [19]. We conducted mating trials from November 2012 to March 2014.

We were unable to standardize the age of spiders at the time of the experiment. The time required to construct a web varied greatly among females, with some females constructing a web within a day and others requiring several weeks. Females without webs still produced enough silk to capture grasshoppers and maintain their feeding regimen during this period. We also could not control time to maturity, so some maturing females did not have adult males immediately available and had to wait until males from the appropriate population matured. Thus, we paired males and females randomly from the pool available, irrespective of age; however, we included time (in days) between molting to adulthood and mating in our statistical models to examine its correlation to courtship and copulation and to control for its effects.

### Statistical analyses

We used χ2 tests to compare treatment groups for both the number of females mating and re-mating and the proportion of males that stayed in the female’s web for four days.

For our experiment on nutritional state, spiders from different collection sites did not differ in their courtship or copulation responses (probability of copulation: Pearson’s test, χ2 = 8.5, df = 4, p = 0.08; number of male courtship bouts: Poisson GLM, χ2 = 2.75, df = 4, p = 0.6; sex initiating courtship: Pearson’s test, χ2 = 16.8, df = 12, p = 0.16). Spiders from all locations were pooled for further analyses.

We tested the effect of our feeding treatments on copulation, cannibalism, and which sex courted first using generalized linear models (GLM) with a binomial distribution and logit link function. We included female feeding regime, male feeding regime, female age, and male age as effects. We included interactions among these effects if they explained a significant amount of the variance. We similarly analyzed the number of male courtships in 30 mins with a GLM, except with a Poisson distribution and a log link function. We take male courtship as a measure of the male’s motivation to mate with the female. We therefore excluded males that did not survive the 30 min trial from this analysis because highly motivated males may have copulated early in the trial, been cannibalized, and thereby prevented from further courtship. We used survivorship analysis (Mantel-Haenszel test) [27] to test the effect of female feeding on the long-term survivorship of the male. Males that died without being cannibalized were censored because they were unlikely to have mated (no female with a male that died of natural causes successfully reproduced). All analyses were conducted in R version 3.1.1.

## Results

### Female re-mating

Females in the three treatments did not differ in their initial propensity to mate on day 0 (χ2 = 0.02, p = 0.988; Table 1). About half of females mated, regardless of their reproductive status (day 0: 52%; re-mating on day 3: 42%; re-mating on day 10: 60%). There was no difference in female mating propensity between any combination of days (0–3 days: χ2 = 0.01, p = 0.917; 0–10 days: χ2 = 0.006, p = 0.937; 3–10 days: χ2 = 0.038, p = 0.843). All males were cannibalized after a single insertion, so no female mated more than twice in the experiment.

**Table 1:** Summary of the female receptivity experiment, presenting the number of successful matings within each group. All females were given males on day 0. In “mated twice” groups, all females were again given males, either on day 3 or on day 10. We show both the total number of females that mated on days 3 and 10 (“Total mating”), as well as just those females that had already mated on day 0 and mated a second time (“Re-mating”).

	Successful mating day 0	Successful mating day 3		Successful mating day 10	
		Total mating	Re-mating	Total mating	Re-mating
Mated once (n = 20)	9	—	—	—	—
Mated twice—day 0 & day 3 (n = 20)	12	9	5	—	—
Mated twice—day 0 & day 10 (n = 20)	10	—	—	12	6

### Male choosiness for female reproductive state

**Fig 2:**
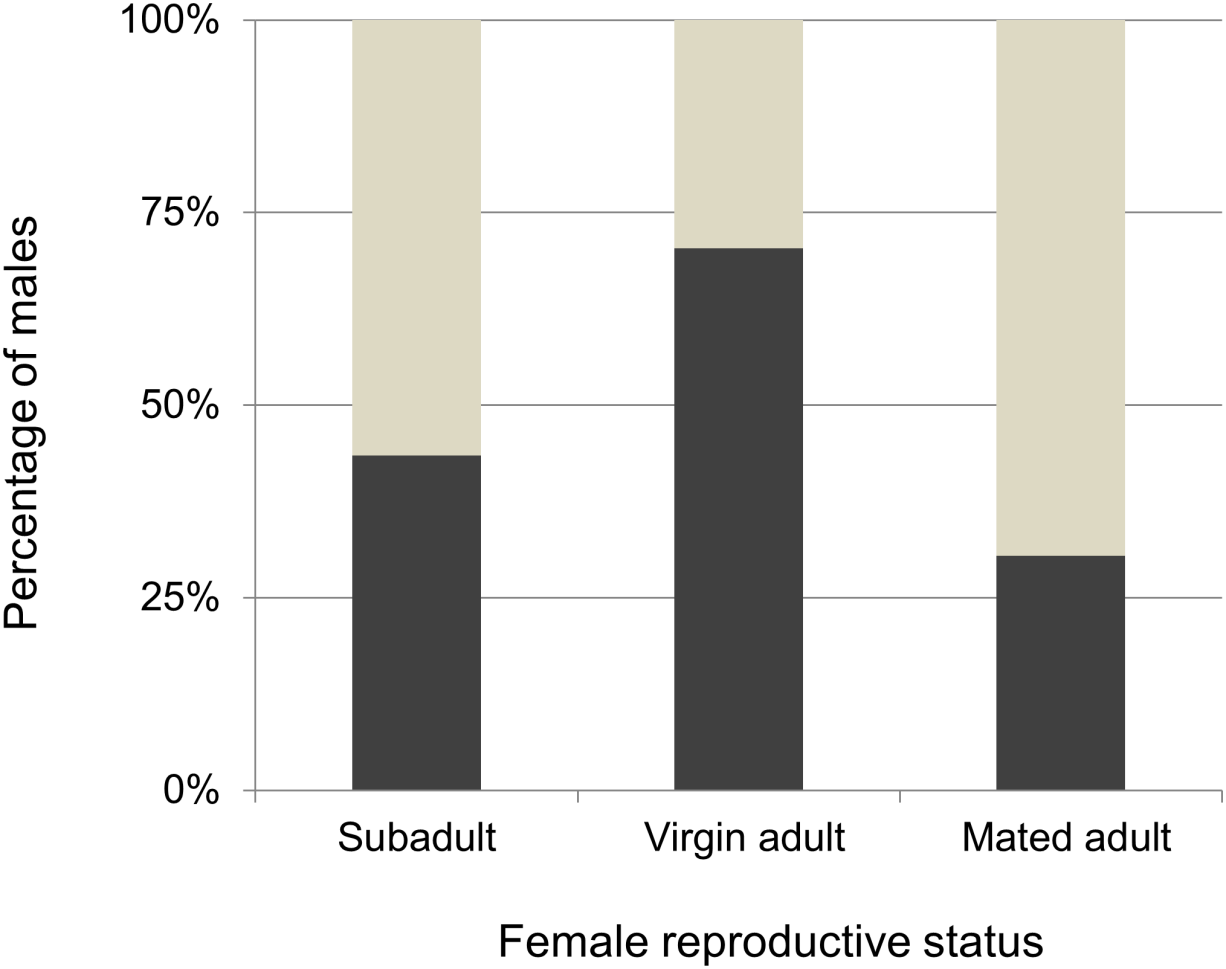
The proportion of males remaining on (dark bars) or dispersing from (grey bars) webs of sub adult, virgin and mated females.

The reproductive status of the females influenced whether males dispersed from the females’ webs (χ2 = 8.382, p = 0.015; Fig 2). Males dispersed more frequently from the webs of both mated (χ2 = 7.935, p = 0.004) and subadult females (χ2 = 3.687, p = 0.05) relative to virgin females. Males dispersed from the webs of mated females and sub-adult females in similar proportion (χ2 = 0.839, p = 0.359).

### Choosiness for mate’s nutritional state and age

**Fig 3:**
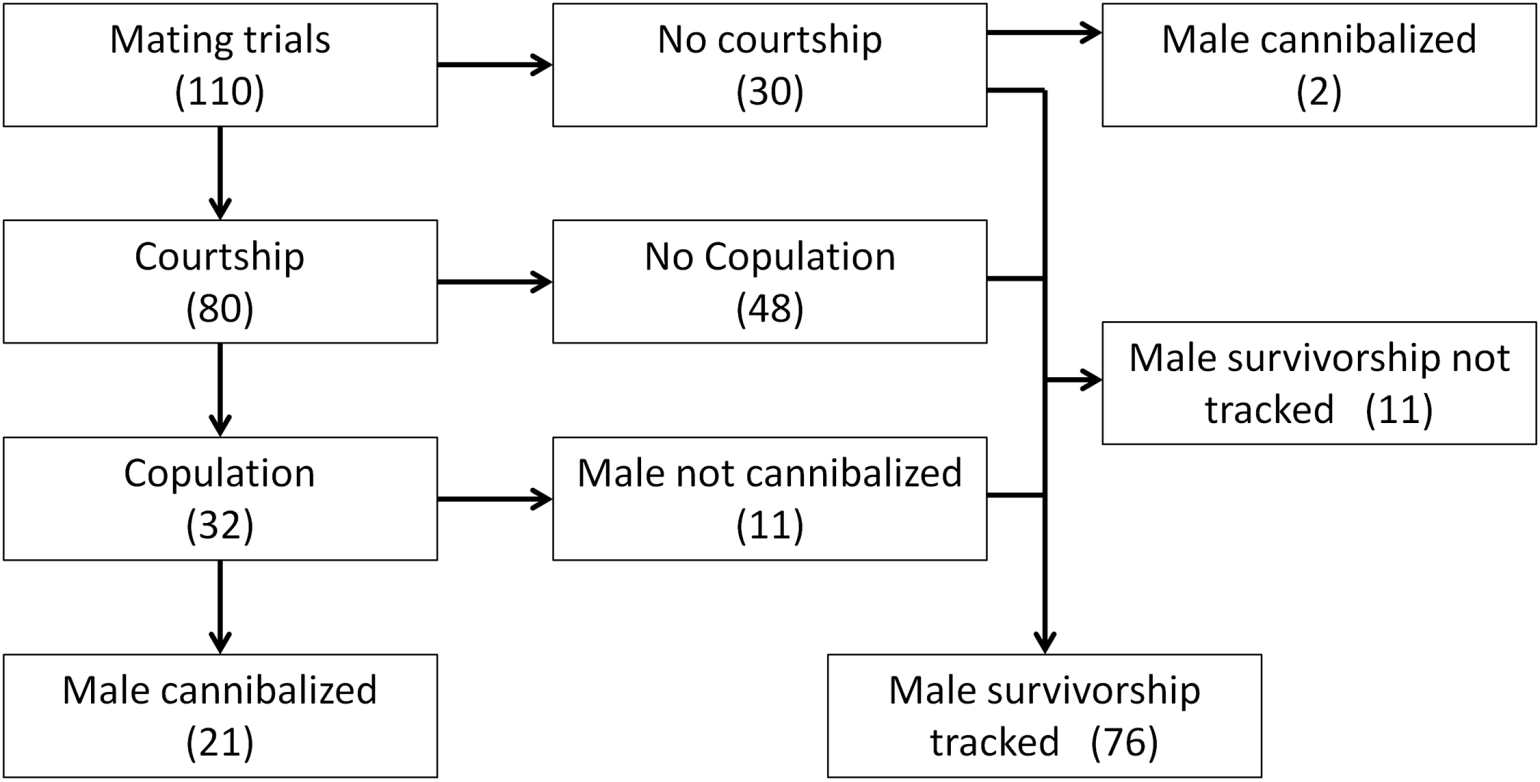
The outcomes of all 110 mating trials that examined male choice for female nutritional state. The trials ended when the male was cannibalized, died in the female’s web, or we ceased tracking male survivorship. Arrows indicate the progression of events, and the numbers of trials are given in parentheses.

**Fig 4:**
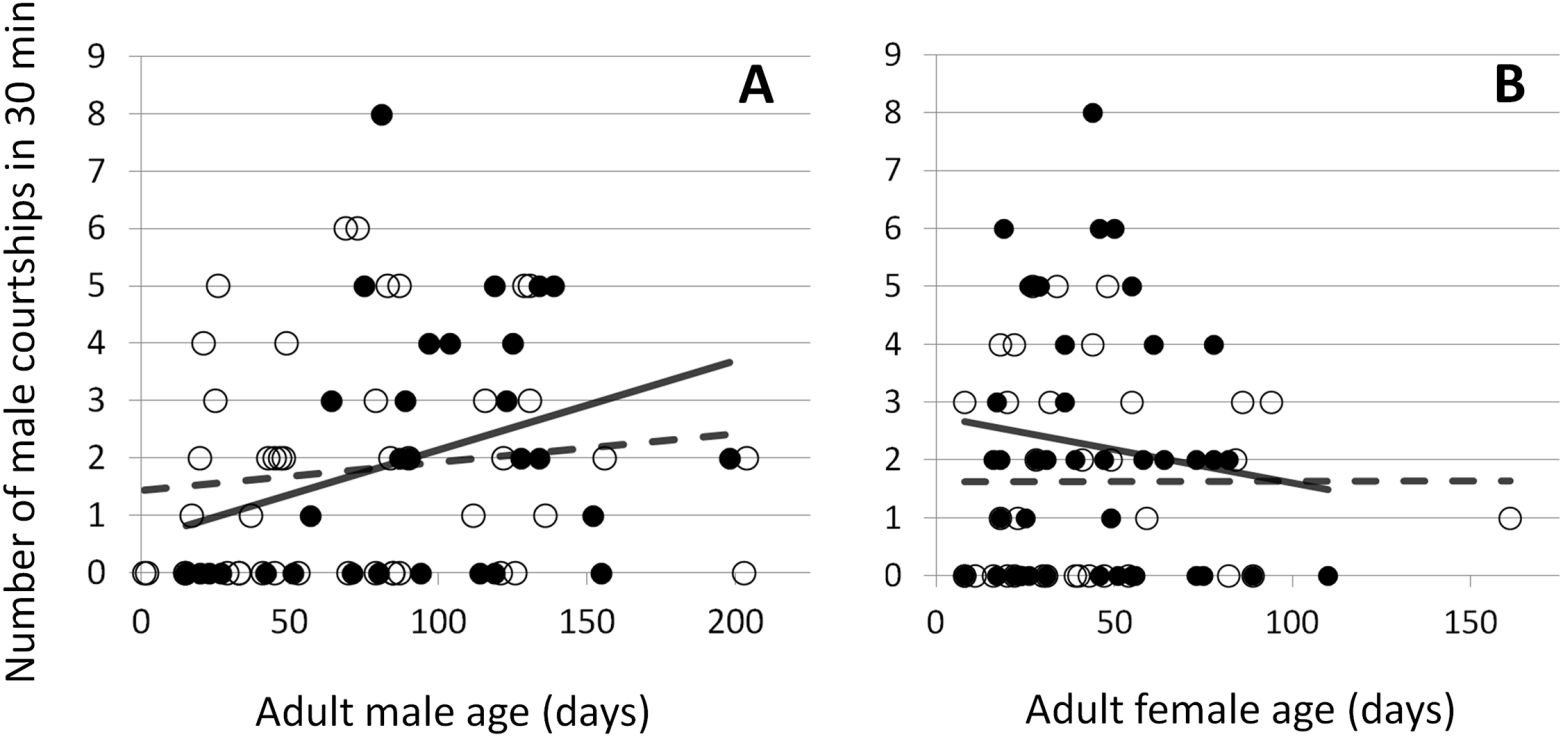
The relationship between the number of male courtships in 30 min and the age of the adult male (A) and female (B). Spiders under the high feeding regime are shown in solid circles with a solid regression line, and spiders under the low feeding regime are shown in open circles with a dashed regression line.

Neither sex courted in 30 (27%) of 110 trials (Fig 3). Of the remaining trials, the male initiated courtship 38 times (48%), and 2 females failed to reciprocate. The female initiated courtship 33 times (41%), and 4 males failed to reciprocate. Both sexes started to court simultaneously in one trial, and we failed to record which sex courted first in 8 trials. Male and female feeding regime and age did not affect which sex courted first (GLM female feeding: z = 0.44, p = 0.66; male feeding: z = 1.47, p = 0.14; female age: z = 1.26, p = 0.21; male age: z = 1.43, p = 0.15). Males courted up to 8 times during the trial. Male courtship decreased with female age and increased with male age (GLM female age: z = 2.1, p = 0.04; male age: z = 3.5, p = 0.0004; Fig 4). There was an interaction between male age and male feeding so that, as high feeding males aged, they increased their courtship more than low feeding males (GLM male feeding x male age: z = 2.2, p = 0.03; Fig 4). There was a non-significant trend for males on the low feeding regime irrespective of age to increase courtship (GLM male feeding: z = 1.8, p = 0.07), and there was no effect of female feeding on male courtship (GLM female feeding: z = 1.4, p = 0.16).

Of the 80 pairs that courted, 32 (40%) copulated within 30 min (Fig 3). During courtship, the female approaches the courting male until she is about 1 cm away. At this point the male makes a final, fast approach to the female (Fig 1A). It was at this final approach that courtship most often failed. There were 197 male courtships across all trials. Of these, 59 (30%) were terminated prior to the final approach. Of the 138 final approaches, 109 (79%) resulted in the male contacting the female but then retreating from her without inserting his pedipalp.

**Fig 5:**
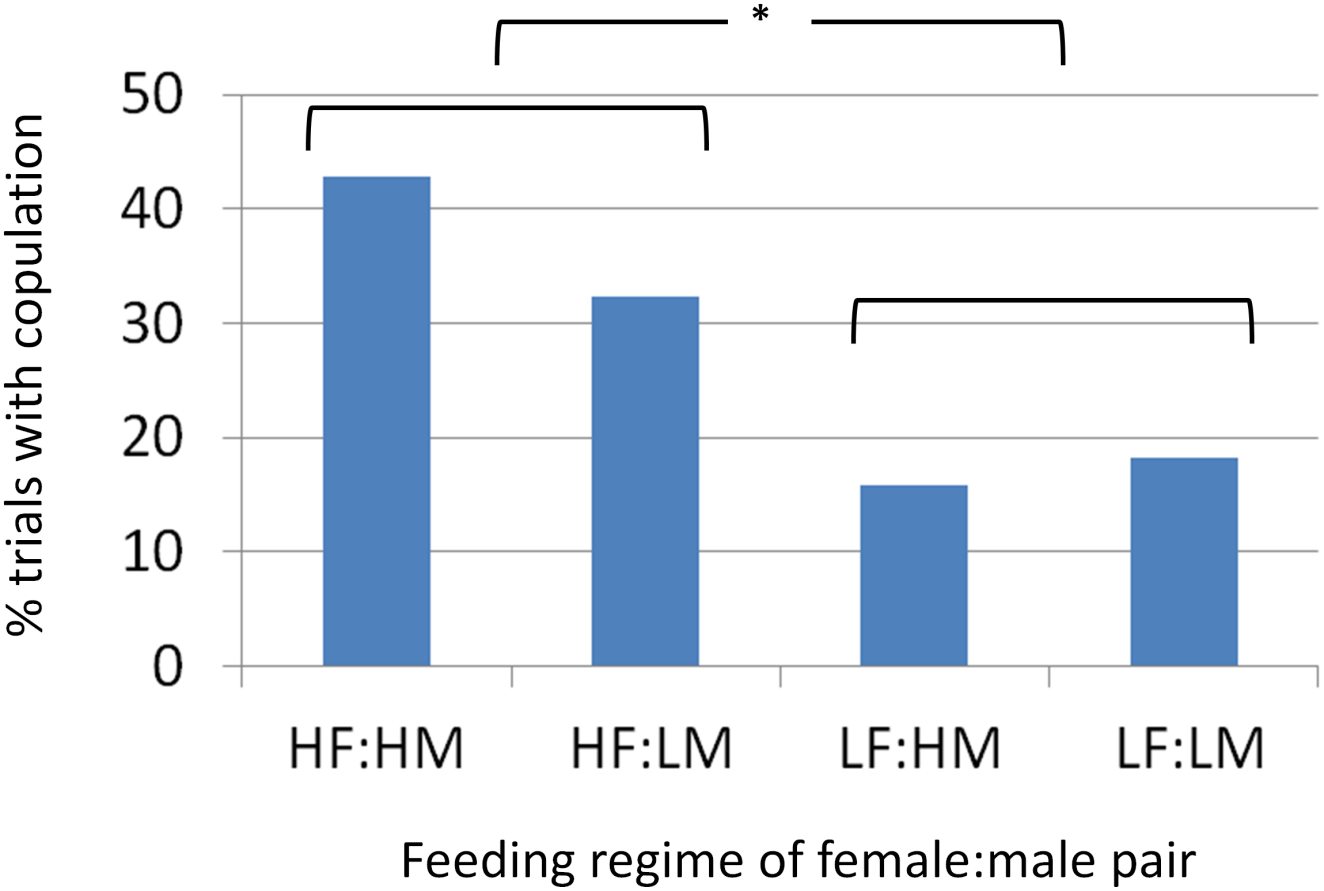
The percentage of trials resulting in copulation separated by feeding regimes. “F” and “M” indicate female and male, respectively, and the preceding letter indicates high “H” or low “L” feeding regimes. Brackets group high and low feeding females, which differed in their copulation probability.

Females on the high feeding regime were twice as likely to copulate as those on the low feeding regime: 24 of 66 (36%) high feeding females and 8 of 44 (18%) low feeding females copulated (GLM female feeding: z = 2.4, p = 0.02). There was no effect of male feeding regime or age of either sex on copulation (GLM male feeding: z = 1.4, p = 0.16; female age: z = 1.3, p = 0.20; male age: z = 0.6, p = 0.56; Fig 5).

**Fig 6:**
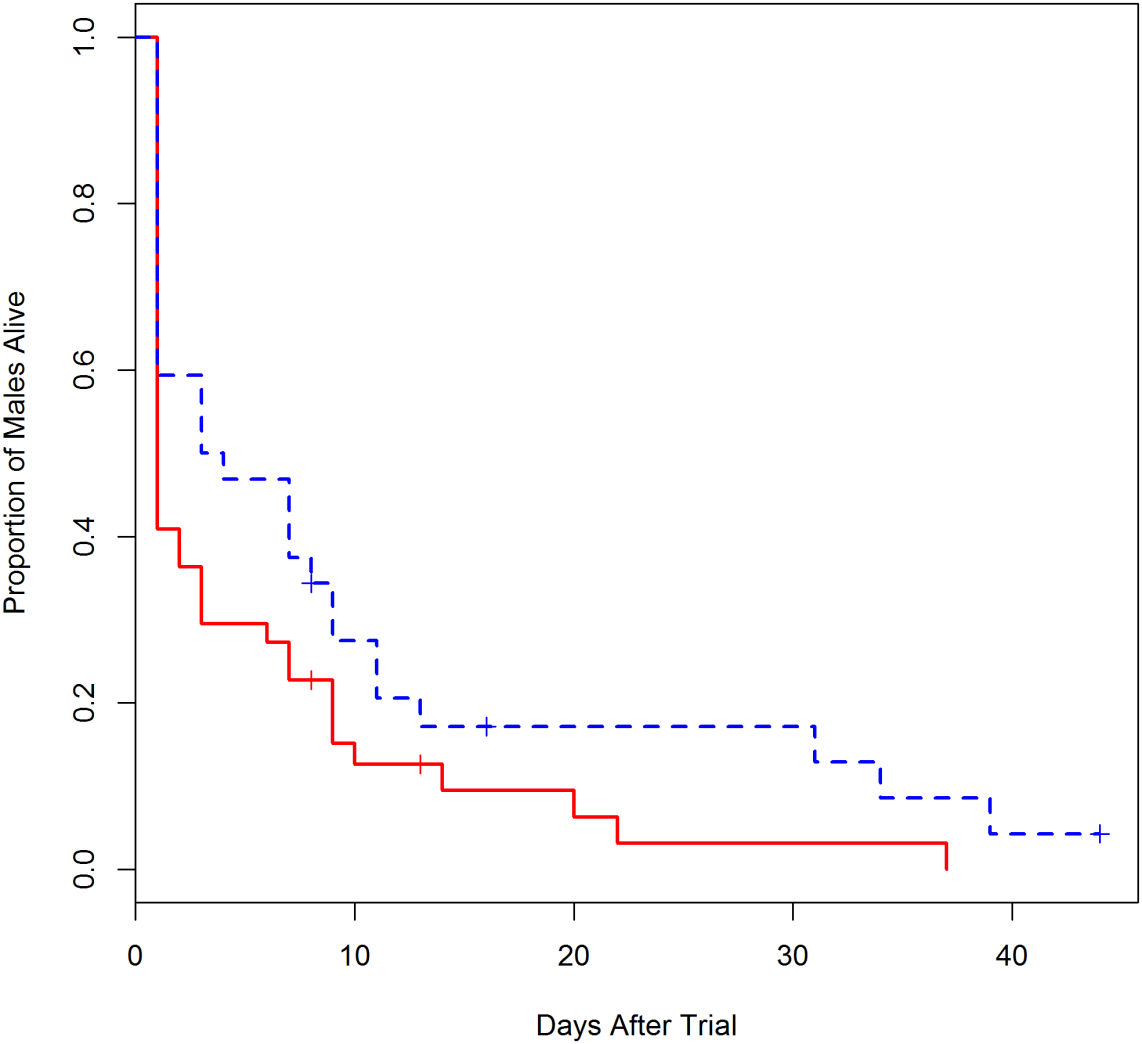
The survivorship curves of males that survived the 30 min trial. The dashed line represents males with low feeding females and the solid line represents males with high feeding females. Crosses indicate males that died but were not cannibalized.

Of the 32 males that copulated, 21 were cannibalized (Fig 3). Seventeen males were cannibalized after one copulation, and 4 were cannibalized after two. All 11 males that survived mating mated only once with the female. Males that survived copulation were more likely to be on the low feeding regime (8/11 were low fed males; GLM male feeding: z = 2.1, p = 0.04). Male and female age had no effect on the probability of cannibalism (GLM: female age: z = 0.57, p = 0.57; male age: z = 0.91, p = 0.36), nor did female feeding (high feeding females cannibalized 67% of their males and low feeding females cannibalized 63%; GLM female feeding: z = 0.68, p = 0.50). We tracked the survivorship of 76 males that survived the mating trials (11 surviving males were not tracked; Fig 3). Males remained alive with the female for up to 44 days. Males with well-fed females (n = 44) tended to die sooner compared to males with poorly-fed females (n = 32), but the difference was not significant (Mantel-Haenszel test: χ2 = 3.3, p = 0.07; Fig 6). The trend was driven primarily by steeper decline in survivorship of males with high feeding regime females one day after the mating trial (Fig 6). In addition to the 21 cases of post-copulatory cannibalism, two males were cannibalized before copulation.

## Discussion

We examined mating behavior in a sexually cannibalistic spider that lives in colonies, where males can frequently encounter multiple, receptive females simultaneously. We predicted that the high rate of post-copulatory sexual cannibalism combined with colonial living should lead to the evolution of male choosiness.

We first tested the possibility of polyandry and found that females were just as likely to mate with a second male as they were with a first, showing that females can mate at least twice. Males, however, usually copulate only once, although the rate of cannibalism after one mating varied between our experiments. Seventeen of 32 males (53%) were cannibalized after a single mating in our experiment on nutritional state, while all 52 males were cannibalized after a single mating in our polyandry experiment. In a previous study, 90% (18/20) of first copulations ended in cannibalism [19]; thus the total body of evidence indicates over 80% of males copulate only once, with an additional 6% copulating twice with the same female. This should lead to an increasingly female-biased sex ratio, particularly as the breeding season progresses and males continue to drop out of the population. While in some other spider species, males are short-lived and may even cease feeding as adults [28], virgin *C*. *citricola* males can live up to ten months [25], so it is cannibalism itself that influences the sex ratio. Primary sex ratios are 1:1 in *C*. *citricola* [25]. Males mature 5–8 instars before females [25], which might lead to more males than females surviving to adulthood [29]. Thus, the operational sex ratio might be male biased initially, with a gradually increasing female bias as females mature and males are cannibalized, which is the case in the sexually cannibalistic *Argiope bruennichi* [30]. Because males are small and easily mistaken for juveniles, we have no reliable measures of tertiary sex ratios in the field. However, because most males are monogamous, a male needs only the expectation of encountering more than one female for choosiness to evolve, assuming that females differ in quality [11]. Because female fecundity increases with caloric intake [25] and prey capture varies within colonies [31], males likely encounter a range of female quality in any given colony.

We tested whether males choose females on the basis of reproductive status, nutritional status, and age. In the net house, we found that males preferred webs of virgin females over those of subadult or mated females. Male spiders commonly choose females on the basis of reproductive state [12, 26, 32]. Wasting reproductive effort on unreceptive or mated females can be very costly, and males of other species sometimes sire no offspring from previously mated females [21]. In the laboratory, we found that males courted more with younger females and that older males increased courtship with all females regardless of female age or feeding regime. The increase in courtship by older males conforms to the common pattern that the choosy sex relaxes choice over time [33], probably due to time constraints on future copulations [23]. Male spiders sometimes select older females to reduce the time between copulation and egg laying [34, 35]. In these species, the reproductive season is short [30, 36], while in *C*. *citricola*, adult females can live over a year [25], produce chains of multiple egg sacs and may repeatedly dump unfertilized eggs if left unmated. Male *C*. *citricola* may prefer young females because they have not yet expended their reproductive resources, as is the case in many other animals [37].

Females on the high feeding regime were twice as likely to copulate as those on the low feeding regime. This result could indicate either that males prefer well-fed females or that well-fed females had a higher propensity to mate. A close inspection of the data suggests the former explanation is more likely. Nearly all females were receptive to male courtship (only two failed to reciprocate), so a lack of response by the female cannot explain the difference between high and low fed females. Copulation failed most often at the final approach of the male (Fig 1A). This is the final component of courtship, so the male makes the ultimate decision to mate or not. Exactly what happens in the brief moment that the male makes contact with the female but does not insert his pedipalp requires further investigation. Although males might prefer well-fed females because they produce more eggs [25], they may also select females that are less hungry and thus less likely to cannibalize them. The data refute this latter explanation because females on the low and high diets cannibalized males at equal rates.

Together, our data support the hypothesis that cannibalism and group living can support the evolution of male choice; however, it is also clear that group living is not required for male choice to evolve. The redback widow (*Latrodectus hasselti*), golden orb-weaver (*Nephila plumipes*), and wasp spider (*Argiope bruennichi*) are models of sexual selection, all with high rates of sexual cannibalism (65%, 60%, 70%, respectively) [38, 39, 40]. They may have clumped distributions, but none to the degree of *C*. *citricola* [30, 41], which makes them informative foils to *C*. *citricola* in understanding how the cost of mating and female encounter rates combine to influence mate choice. The cost of mate search is extreme in *L*. *hasselti*, with 80% of males perishing before locating a mate [38]. Males prefer virgin to mated females, but preference for female size or condition has not yet been found [12]. *Nephila plumipes* males also face high search costs (64% of males fail to find a female), and males similarly prefer penultimate over adult females but have no preference for larger and more fecund females [42, 43]. Males of *A*. *bruennichi*, by contrast, do choose females on both the basis of virginity and fecundity, but the decisions are complex. Males seem to employ a trading up strategy: most males copulate with the first female they encounter, but a few males may switch to larger females [44], and males abandon their first mate more often if she is young and small [35]. Older males show a preference for larger females that younger males lack [45], which contrasts with the decreasing choosiness with male age found in *C*. *citricola*. Search costs in *A*. *bruennichi* have not yet been characterized to our knowledge. While males in the above studies could choose among two or more mating options, we presented *C*. *citricola* males with a single female. No male in our studies survived two copulations with the same female, suggesting that the many males that survived after our mating trials refrained from mating with virgin females for long periods of time, even with no other options available (Fig 6). Future work should present *C*. *citricola* males with a choice between females to more directly compare male choosiness to other systems. In addition, search costs for *C*. *citricola* males remain unknown, but we suspect they will be relatively low due to their colonial lifestyle.

Choosiness on the basis of female reproductive state is common, even when search costs are high, but male choosiness in this context will not generate sexual selection on females [6]. More subtle discrimination by males, based on nutritional state or fecundity, is less common [32] but will impose sexual selection on females to increase their chances of finding a mate, perhaps through more aggressive courtship, as seen in *C*. *citricola*. It was surprising that *C*. *citricola* females initiated courtship nearly as often as males. In nearly all spiders, males initiate vibratory courtship [46], which is often triggered by contact pheromones on the female’s web [26, 47]. It was also possible that some females that failed to court were unaware of the male’s presence. Neither sex courted in 27% of trials, and in many of these cases the male did not move for 30 mins, which may have rendered him difficult to detect. It is unlikely that the male was unaware of the female because our data show that males recognize female webs and assess female reproductive status by silk cues alone. Male courtship is thought to signal species identity (and halt the female’s predatory response) and male quality [46]. The readiness of females to court with minimal information about the male aligns with our failure to detect female choosiness on the basis of male feeding history. Our data suggest that, not only are males choosy, but that they are also more choosy than females, indicating a sex-role reversal.

The high rate of sexual cannibalism in *C*. *citricola* raises the question whether males are complicit in their own demise. In some spiders with high rates of sexual cannibalism (e.g. *Araneus pallidus*, *Latrodectus hasselti*, and *L*. *geometricus*), the male summersaults into the female’s chelicerae during copulation [48]. *Cyrtophora citricola* males perform no similar maneuver. Instead, the female bends her cephalothorax toward her abdomen to bite the male (Fig 1C), and the orientation of the male relative to the female (Fig 1B) ensures a successful attack. The male makes no attempt to change his orientation during copulation to enable an escape, suggesting that the male may derive some benefit from being cannibalized. According to theory, male complicity in sexual cannibalism can evolve as a means of securing paternity in the face of sperm competition [15, 49]. Berner-Aharon [19], found no evidence of mating plugs in the genitalia of female *C*. *citricola*. Most spider mating plugs derive from mutilated male genitalia, but some are secretions by either the male or female [50], which might have been undetectable by microscope. Because increased paternity accompanies sexual cannibalism in other spider species [39, 40, 51], we predict that further investigation will reveal some paternity benefit of male sacrifice in *C*. *citricola*, if not a cryptic mating plug, then perhaps longer copulation time or increased sperm transfer [51].

Our data show that post-copulatory cannibalism in *C*. *citricola* enforces monogamy in most males, that males choose females on the basis of reproductive state, age, and nutritional state, and that females usually accept males and sometimes initiate courtship. These findings suggest a co-evolutionary cycle: cannibalism by females led to mate choice by males, which may, in turn, select for more aggressively courting females. The role of sexual cannibalism in male mate choice and sex-role reversal is still not fully understood [12], and the role of male choosiness in female evolution has rarely been investigated. Much work remains to be done to understand how *C*. *citricola* fits into other systems, but the spider genus *Cyrtophora*, comprising both group-living and solitary species, may be an interesting system in which to investigate the combined effects of sexual cannibalism and mate availability on sexual selection for male choosiness.

## Supporting Information

S1 Raw Data File contains all data used in this manuscript. (XLSX)
